# Supplementary material for: Crawling and Gliding: A Computational Model for Shape-Driven Cell Migration
Source: PLoS Comput Biol. 2015 Oct 21;11(10):e1004280. doi: 10.1371/journal.pcbi.1004280 (PMC4619082; doi:10.1371/journal.pcbi.1004280)
Supplement: S1 Code — (ZIP) [file pcbi.1004280.s012.zip › release/tst/doc/html/classCellularPotts.html]

Tissue Simulation Toolkit: CellularPotts Class Reference


Tissue Simulation Toolkit
 0.1.4.1


- Main Page
- Namespaces
- Classes
- Files

- Class List
- Class Hierarchy
- Class Members

Public Member Functions |
Public Attributes |
Protected Member Functions |
Protected Attributes |
Friends |
List of all members

CellularPotts Class Reference

`#include <ca.h>`

|  |  |
| --- | --- |
| Public Member Functions | |
|  | CellularPotts (std::vector< Cell > \*cells, const int sizex=200, const int sizey=200) |
|  | Constructs a CA field. This should be done in "Dish". More... |
|  | |
|  | CellularPotts (void) |
|  | |
| virtual void | AllocateSigma (int sx, int sy) |
|  | |
| virtual | ~CellularPotts () |
|  | |
| int \*\* | SearchNandPlot (Graphics \*g=0, bool get\_neighbours=true) |
|  | Plots the dish to the screen or to a movie and searches the neighbours. More... |
|  | |
| void | Plot (Graphics \*g) |
|  | Plot the dish to Graphics window g. More... |
|  | |
| int \*\* | SearchNeighbours (void) |
|  | Searches the cells' neighbors without plotting. More... |
|  | |
| int | Mass (void) |
|  | Return the total area occupied by the cells. More... |
|  | |
| void | PlotSigma (Graphics \*g, int mag=2) |
|  | |
| void | DivideCells (void) |
|  | Divide all cells. More... |
|  | |
| void | DivideCells (std::vector< bool > which\_cells) |
|  | |
| int | AmoebaeMove (PDE \*PDEfield=0) |
|  | Monte Carlo Step. Returns summed energy change. More... |
|  | |
| void | ReadZygotePicture (void) |
|  | Read initial cell shape from XPM file. Reads the initial cell shape from an include xpm picture called "ZYGXPM(ZYGOTE)", and it allocates enough cells for it to the Dish. More... |
|  | |
| void | ConstructInitCells (Dish &beast) |
|  | |
| int | Time () const |
|  | Returns the number of completed Monte Carlo steps. More... |
|  | |
| int | ZygoteArea () const |
|  | |
| int | SizeX () const |
|  | Return the horizontal size of the CA plane. More... |
|  | |
| int | SizeY () const |
|  | Return the vertical size of the CA plane. More... |
|  | |
| int | Sigma (const int x, const int y) const |
|  | Return the value of lattice site (x,y). More... |
|  | |
| void | Replace (Graphics \*g) |
|  | |
| Dir \* | FindCellDirections (void) const |
|  | |
| int | ThrowInCells (int n, int cellsize) |
|  | Initialize the CA plane with n circular cells fitting in a cellsize^2 square. More... |
|  | |
| int | GrowInCells (int n\_cells, int cellsize, double subfield=1.) |
|  | Initialize the CA plane with n cells using an Eden growth algorithm. More... |
|  | |
| int | GrowInCells (int n\_cells, int cell\_size, int sx, int sy, int offset\_x, int offset\_y) |
|  | |
| Cell & | AddCell (Dish &beast) |
|  | Adds a new Cell and returns a reference to it. More... |
|  | |
| void | ShowDirections (Graphics &g, const Dir \*celldir) const |
|  | Display the division planes returned by FindCellDirections. More... |
|  | |
| double | MeanCellArea (void) const |
|  | Returns the mean area of the cells. More... |
|  | |
| double | CellDensity (void) const |
|  | Returns the cell density. More... |
|  | |
| void | ResetTargetLengths (void) |
|  | Set target lengths of all cells to the value given in parameter file. More... |
|  | |
| void | SetRandomTypes (void) |
|  | Give each cell a random cell type. More... |
|  | |
| void | GrowAndDivideCells (int growth\_rate) |
|  | |
| Cell & | getCell (int c) |
|  | |
| double | DrawConvexHull (Graphics \*g, int color=1) |
|  | |
| double | Compactness (double \*res\_compactness=0, double \*res\_area=0, double \*res\_cell\_area=0) |
|  | |

|  |  |
| --- | --- |
| Public Attributes | |
| int | spins\_converted |
|  | |

|  |  |
| --- | --- |
| Protected Member Functions | |
| void | BaseInitialisation (std::vector< Cell > \*cell) |
|  | |

|  |  |
| --- | --- |
| Protected Attributes | |
| int \*\* | sigma |
|  | |
| int | sizex |
|  | |
| int | sizey |
|  | |

|  |  |
| --- | --- |
| Friends | |
| class | Info |
|  | |
| class | Morphometry |
|  | |

## Constructor & Destructor Documentation

|  |  |  |  |
| --- | --- | --- | --- |
| CellularPotts::CellularPotts | ( | std::vector< Cell > \* | *cells*, |
|  |  | const int | *sizex* = `200`, |
|  |  | const int | *sizey* = `200` |
|  | ) |  |  |

Constructs a CA field. This should be done in "Dish".

|  |  |  |  |  |  |
| --- | --- | --- | --- | --- | --- |
| CellularPotts::CellularPotts | ( | void |  | ) |  |

References Parameter::neighbours, and Parameter::T.

|  |  |  |  |  |  |
| --- | --- | --- | --- | --- | --- |
| CellularPotts::~CellularPotts | ( | void |  | ) |  |

virtual

## Member Function Documentation

|  |  |  |  |  |  |
| --- | --- | --- | --- | --- | --- |
| Cell& CellularPotts::AddCell | ( | Dish & | *beast* | ) |  |

inline

Adds a new Cell and returns a reference to it.

|  |  |  |  |
| --- | --- | --- | --- |
| void CellularPotts::AllocateSigma | ( | int | *sx*, |
|  |  | int | *sy* |
|  | ) |  |  |

virtual

References MemoryWarning().

|  |  |  |  |  |  |
| --- | --- | --- | --- | --- | --- |
| int CellularPotts::AmoebaeMove | ( | PDE \* | *PDEfield* = `0` | ) |  |

Monte Carlo Step. Returns summed energy change.

Implements the core CPM algorithm. Carries out one MCS.

Returns
:   Total energy change during MCS.

References Parameter::conn\_diss, Parameter::periodic\_boundaries, and RANDOM().

|  |  |  |  |  |  |
| --- | --- | --- | --- | --- | --- |
| void CellularPotts::BaseInitialisation | ( | std::vector< Cell > \* | *cell* | ) |  |

protected

References Parameter::neighbours, and Parameter::T.

|  |  |  |  |  |  |
| --- | --- | --- | --- | --- | --- |
| double CellularPotts::CellDensity | ( | void |  | ) | const |

Returns the cell density.

Cell density is defined as the area occupied by cells divided by the size of the field.

|  |  |  |  |
| --- | --- | --- | --- |
| double CellularPotts::Compactness | ( | double \* | *res\_compactness* = `0`, |
|  |  | double \* | *res\_area* = `0`, |
|  |  | double \* | *res\_cell\_area* = `0` |
|  | ) |  |  |

Calculate compactness (summed\_area/hull\_area) of all cells. This is a good measure for the density.

Returns
:   Compactness.

References Cell::Area(), chainHull\_2D(), Point::x, and Point::y.

|  |  |  |  |  |  |
| --- | --- | --- | --- | --- | --- |
| void CellularPotts::ConstructInitCells | ( | Dish & | *beast* | ) |  |

References Parameter::target\_area.

|  |  |  |  |  |  |
| --- | --- | --- | --- | --- | --- |
| void CellularPotts::DivideCells | ( | void |  | ) |  |

inline

Divide all cells.

|  |  |  |  |  |  |
| --- | --- | --- | --- | --- | --- |
| void CellularPotts::DivideCells | ( | std::vector< bool > | *which\_cells* | ) |  |

Divide all cells marked "true" in which\_cells.

Parameters
:   |  |  |
    | --- | --- |
    | which\_cells | is a vector<bool> with the same number of elements as the number of cells. It is a mask indicating which cells should be divided; each cell marked true will be divided. |

If which\_cells is empty, this method divides all cells.

|  |  |  |  |
| --- | --- | --- | --- |
| double CellularPotts::DrawConvexHull | ( | Graphics \* | *g*, |
|  |  | int | *color* = `1` |
|  | ) |  |  |

Draw convex hull around all cells.

Returns
:   The area of the convex hull in lattice sites.

References chainHull\_2D(), Graphics::Line(), Point::x, and Point::y.

|  |  |  |  |  |  |
| --- | --- | --- | --- | --- | --- |
| Dir \* CellularPotts::FindCellDirections | ( | void |  | ) | const |

In this method the principal axes of the cells are computed using the method described in "Biometry", box 15.5

Returns
:   a pointer to a "new[]"ed array containing the directions. The memory has to be freed afterwards using the delete[] operator

References Dir::aa1, Dir::aa2, Dir::bb1, Dir::bb2, Dir::lb1, Dir::lb2, and MemoryWarning().

|  |  |  |  |  |  |
| --- | --- | --- | --- | --- | --- |
| Cell& CellularPotts::getCell | ( | int | *c* | ) |  |

inline

|  |  |  |  |  |  |
| --- | --- | --- | --- | --- | --- |
| void CellularPotts::GrowAndDivideCells | ( | int | *growth\_rate* | ) |  |

Cells grow until twice their original target\_length, then divide, with rate "growth\_rate"

References Cell::SetTargetArea(), and Parameter::target\_area.

|  |  |  |  |
| --- | --- | --- | --- |
| int CellularPotts::GrowInCells | ( | int | *n\_cells*, |
|  |  | int | *cellsize*, |
|  |  | double | *subfield* = `1.` |
|  | ) |  |  |

Initialize the CA plane with n cells using an Eden growth algorithm.

Parameters
:   |  |  |
    | --- | --- |
    | n | Number of cells. |
    | cellsize | Number of Eden growth iterations. |
    | subfield | Defines a centered frame of size (size/subfield)^2 in which all cell will be positioned. |

Returns
:   Index of last cell inserted.

|  |  |  |  |
| --- | --- | --- | --- |
| int CellularPotts::GrowInCells | ( | int | *n\_cells*, |
|  |  | int | *cell\_size*, |
|  |  | int | *sx*, |
|  |  | int | *sy*, |
|  |  | int | *offset\_x*, |
|  |  | int | *offset\_y* |
|  | ) |  |  |

References MemoryWarning(), RANDOM(), and RandomNumber().

|  |  |  |  |  |  |
| --- | --- | --- | --- | --- | --- |
| int CellularPotts::Mass | ( | void |  | ) |  |

inline

Return the total area occupied by the cells.

References sigma, sizex, and sizey.

|  |  |  |  |  |  |
| --- | --- | --- | --- | --- | --- |
| double CellularPotts::MeanCellArea | ( | void |  | ) | const |

Returns the mean area of the cells.

References Cell::Area().

|  |  |  |  |  |  |
| --- | --- | --- | --- | --- | --- |
| void CellularPotts::Plot | ( | Graphics \* | *g* | ) |  |

inline

Plot the dish to Graphics window g.

References SearchNandPlot().

|  |  |  |  |
| --- | --- | --- | --- |
| void CellularPotts::PlotSigma | ( | Graphics \* | *g*, |
|  |  | int | *mag* = `2` |
|  | ) |  |  |

Plot the cells according to their cell identity, not their type.

The black lines are omitted.

A simple method to plot all sigma's in window without the black lines

References Graphics::Point().

|  |  |  |  |  |  |
| --- | --- | --- | --- | --- | --- |
| void CellularPotts::ReadZygotePicture | ( | void |  | ) |  |

Read initial cell shape from XPM file. Reads the initial cell shape from an include xpm picture called "ZYGXPM(ZYGOTE)", and it allocates enough cells for it to the Dish.

References Cell::MaxSigma(), MemoryWarning(), ZYGOTE, and ZYGXPM.

|  |  |  |  |  |  |
| --- | --- | --- | --- | --- | --- |
| void CellularPotts::Replace | ( | Graphics \* | *g* | ) |  |

|  |  |  |  |  |  |
| --- | --- | --- | --- | --- | --- |
| void CellularPotts::ResetTargetLengths | ( | void |  | ) |  |

Set target lengths of all cells to the value given in parameter file.

References Cell::SetTargetLength(), and Parameter::target\_length.

|  |  |  |  |
| --- | --- | --- | --- |
| int \*\* CellularPotts::SearchNandPlot | ( | Graphics \* | *g* = `0`, |
|  |  | bool | *get\_neighbours* = `true` |
|  | ) |  |  |

Plots the dish to the screen or to a movie and searches the neighbours.

These distinct tasks have been lumped together in the same method because both for drawing the black lines between the cells and for searching the neighbours the cell borders have to be determined.

References EMPTY, MemoryWarning(), and Graphics::Point().

Referenced by Plot(), and SearchNeighbours().

|  |  |  |  |  |  |
| --- | --- | --- | --- | --- | --- |
| int\*\* CellularPotts::SearchNeighbours | ( | void |  | ) |  |

inline

Searches the cells' neighbors without plotting.

References SearchNandPlot().

|  |  |  |  |  |  |
| --- | --- | --- | --- | --- | --- |
| void CellularPotts::SetRandomTypes | ( | void |  | ) |  |

Give each cell a random cell type.

The number of cell types is defined by the J parameter file. (See Jtable in parameter file).

References Cell::maxtau, and RandomNumber().

|  |  |  |  |
| --- | --- | --- | --- |
| void CellularPotts::ShowDirections | ( | Graphics & | *g*, |
|  |  | const Dir \* | *celldir* |
|  | ) |  | const |

Display the division planes returned by FindCellDirections.

Parameters
:   |  |  |
    | --- | --- |
    | g | Graphics window |
    | celldir | cell axes as returned by FindCellDirections. |

References Dir::aa1, Dir::bb1, and Graphics::Line().

|  |  |  |  |
| --- | --- | --- | --- |
| int CellularPotts::Sigma | ( | const int | *x*, |
|  |  | const int | *y* |
|  | ) |  | const |

inline

Return the value of lattice site (x,y).

i.e. This will return the index of the cell which occupies site (x,y).

References sigma.

Referenced by PDE::Plot().

|  |  |  |  |  |
| --- | --- | --- | --- | --- |
| int CellularPotts::SizeX | ( |  | ) | const |

inline

Return the horizontal size of the CA plane.

References sizex.

|  |  |  |  |  |
| --- | --- | --- | --- | --- |
| int CellularPotts::SizeY | ( |  | ) | const |

inline

Return the vertical size of the CA plane.

References sizey.

|  |  |  |  |
| --- | --- | --- | --- |
| int CellularPotts::ThrowInCells | ( | int | *n*, |
|  |  | int | *cellsize* |
|  | ) |  |  |

Initialize the CA plane with n circular cells fitting in a cellsize^2 square.

! Fill the plane with initial cells

Returns
:   actual amount of cells (some are not draw due to overlap)

References RandomNumber().

|  |  |  |  |  |
| --- | --- | --- | --- | --- |
| int CellularPotts::Time | ( |  | ) | const |

inline

Returns the number of completed Monte Carlo steps.

|  |  |  |  |  |
| --- | --- | --- | --- | --- |
| int CellularPotts::ZygoteArea | ( |  | ) | const |

inline

## Friends And Related Function Documentation

friend class Info

friend

friend class Morphometry

friend

## Member Data Documentation

int\*\* CellularPotts::sigma

protected

Referenced by Mass(), and Sigma().

int CellularPotts::sizex

protected

Referenced by Mass(), and SizeX().

int CellularPotts::sizey

protected

Referenced by Mass(), and SizeY().

int CellularPotts::spins\_converted

---

The documentation for this class was generated from the following files:

- ca.h
- ca.cpp


---

Generated on Thu Aug 14 2014 22:04:01 for Tissue Simulation Toolkit by  

 1.8.6
